# Isolated involvement of corpus callosum in metronidazole-induced encephalopathy with concomitant peripheral neuropathy: A case report

**DOI:** 10.1097/MD.0000000000020198

**Published:** 2020-05-15

**Authors:** Qing Peng, Qian You, Jing Zhang, Shui Liu

**Affiliations:** a Department of Neurology; b Department of Radiology, Peking University First Hospital, Beijing, China.

**Keywords:** metronidazole, metronidazole-induced encephalopathy, peripheral neuropathy, reversible splenial lesion syndrome

## Abstract

**Rationale:**

Metronidazole is widely used for treating infection of anaerobic bacteria and protozoa. Metronidazole is generally well tolerated, although metronidazole-associated peripheral neuropathy (PN) and metronidazole-induced encephalopathy (MIE) have been reported as rare side effects. The most common sites of MIE involve the bilateral dentate nucleus of the cerebellum. Herein, we present a rare case of MIE with isolated corpus callosum involvement, with concomitant metronidazole-associated PN.

**Patient concerns:**

A middle-aged man with ulcerative colitis was diagnosed with amoebic dysentery because of unhygienic eating. After receiving metronidazole (1.8 g/d, cumulative dose 61.2 g) for >1 month, he started to complain of continuous paresthesia of the limbs, and intermittent speech problems. Magnetic resonance imaging demonstrated an isolated lesion in the splenium of the corpus callosum.

**Diagnosis:**

A diagnosis of reversible splenial lesion syndrome and PN was made. Given the patient's medical history, MIE and metronidazole-associated PN were considered.

**Interventions:**

Metronidazole was stopped. Mecobalamine and vitamin B1 were used for adjuvant treatment.

**Outcomes:**

At 1.5 months after stopping metronidazole, his symptoms of numbness and hyperesthesia had not improved, although he felt less ill. The isolated lesion disappeared on follow-up magnetic resonance imaging. At 6 months later, the hyperesthesia symptoms remained, and he was unable to resume his previous work.

**Conclusions:**

Physicians should consider MIE in their differentials for reversible splenial lesion syndrome when encountering a patient with a history of metronidazole medication and symptoms of encephalopathy, especially with concomitant PN. Early identification of this metronidazole-related complication and early cessation of the drug are essential for treatment.

## 1 Introduction

Metronidazole is widely used for its potent effect against anaerobic bacteria and protozoa.^[1]^ It is generally well tolerated, but in rare cases, serious neurotoxicity, including peripheral neuropathy (PN) and metronidazole-induced encephalopathy (MIE), has been reported.^[2,3]^ Previous studies have suggested that after cessation of metronidazole, the majority of patients with MIE or metronidazole-associated PN will partially or fully recover.^[4]^ Thus, early diagnosis of this condition and early cessation of the drug is essential to treatment. However, because of the scarcity of related studies, more clinical evidence is required to elucidate the mechanism of metronidazole-associated neurotoxicity. Herein, we present a rare case of MIE with isolated corpus callosum involvement, with concomitant metronidazole-associated PN.

### 1.1 Case presentation

A 46-year-old man visited our clinic complaining fever, anorexia, diarrhea, and jam-like stool for 3 months after eating undercooked wontons. For past medical history, the patient developed intermittent diarrhea more than 2 years prior, but did not receive formal hospital treatment. After admission, amoebic cysts and trophozoites were detected from a stool smear, and he was diagnosed with amoebic dysentery. He was treated with intravenous infusion of ornidazole and etimicin for 9 days, followed by oral metronidazole for <1 month (1.8 g/d, cumulative dose 61.2 g), after which his symptoms of diarrhea largely resolved. Follow-up colonoscopy showed Epstein–Barr virus infection in the colon, pseudomembranous colitis, amoebic bowel disease, and ulcerative colitis. Given these findings, meropenem and mesalazine were then prescribed.

**Figure 1:**
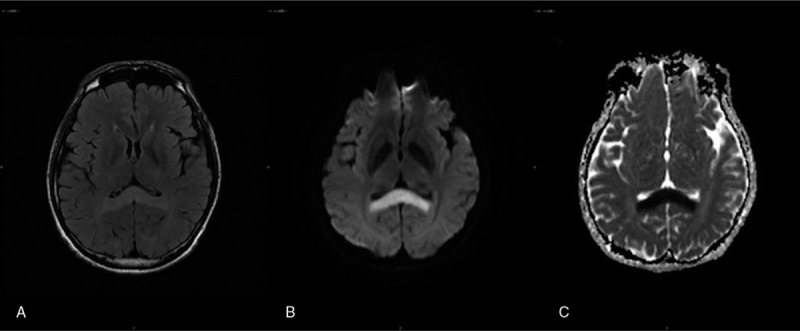
MRI on the day the patient felt difficulty with speech. (A) T2- FLAIR imaging showed hyperintense lesions in the splenium of corpus callosum (white arrow). (B) DWI showed hyperintensity in the corresponding area (white arrow). (C) ADC revealed hypointense lesions in the corresponding area (white arrow). MRI = magnetic resonance imaging; FLAIR = fluid attenuation inversion recovery  ; DWI = diffusion weighted imaging  ; ADC = apparent diffusion coefficient  .

At approximately 1 month after initiating metronidazole treatment, he started to feel numbness in his finger tips and planta pedis bilaterally, without any limb weakness. The numbness gradually progressed from his feet to knee joints. At the same time, his hands became swollen and tingled upon pressure. Further, his feet became edematous and tingled when walking, and he occasionally went to bed with his shoes on without realizing. He also experienced an episode of being stuck in a speech problem for approximately 2 h, which then spontaneously recovered without any repeated attacks. During the episode, he could only say simple words such as ‘mama’, while his ability to understand what others said was totally intact. Blood glucose, among many other laboratory findings, was normal during the whole episode. Peripheral nerve conduction velocity showed severe sensory dominant axonopathy. T2-weighted fluid attenuation inversion recovery imaging revealed hyperintense lesions in the splenium of the corpus callosum, while diffusion weighted imaging (DWI) showed a high signal in the corresponding lesion (Fig. 1). Diagnosis of reversible splenial lesion syndrome (RESLES) and PN was made.

Given the patient's medicine history, MIE and metronidazole-associated PN was considered. Therefore, metronidazole was discontinued and he was transferred to our neurology department for further treatment. Bilateral feet edema were noted on physical examination. Neurological examination revealed glove-and-socking-like hypalgesia and hypopselaphesia, as well as socking-like hyperpathia. Vibration sensation of the bilateral lower extremities was decreased. The power of gripping of the hand and plantar flexion of the foot were weakened, which we assume was caused by pain when exerting strength. Tendon reflexes and coordination movements were normal. Pathological signs were negative.

**Figure 2:**
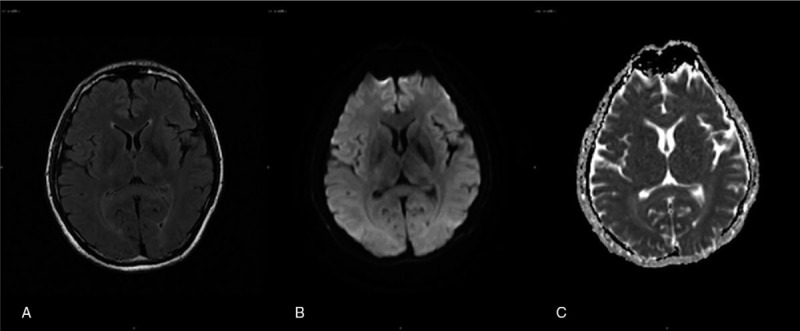
Follow-up MRI after cessation of metronidazole for 1.5 mo. (A) The abnormal findings on T2-weighted FLAIR imaging completely disappeared. (B) The abnormal findings on DWI disappeared. (C) The abnormal findings on ADC disappeared. ). MRI = magnetic resonance imaging; FLAIR = fluid attenuation inversion recovery  ; DWI = diffusion weighted imaging  ; ADC = apparent diffusion coefficient  .

Mecobalamine and vitamin B1 were used for adjuvant treatment. At 1.5 months after cessation of metronidazole, his paresthesia improved slightly, but there was no obvious overall improvement in his condition. The lesion disappeared on follow-up magnetic resonance imaging (MRI) (Fig. 2). After a further 6 months, the symptom of hyperesthesia remained, and he could not resume his previous work normally. The clinical and imaging data were obtained with the patient's consent for publication of this report.

## 2 Discussion

Reversible lesions involving the splenium of the corpus callosum have been previously reported as RESLES, and are a rare comorbidity of multiple diseases. The manifestation is non-specific, typically including headache and consciousness disturbance, and is mainly caused by drugs (especially antiepileptic drugs), viral infection and metabolic disorders, especially hypoglycemia and hypernatremia,^[5,6]^. The prognosis is always good, with no remaining neurological deficits after etiological treatment. The characteristic imaging features involve a localized elliptical or strip-like lesion on the splenium of the corpus callosum. If there is a strip-like lesion involving the entire corpus callosum, it is termed the ‘boomerang sign’, as observed in our case. Thus, our case was diagnosed with RESLES.

The possible etiology included drugs (particularly metronidazole), infection (EBV or amoeba), and metabolic disturbance caused by diarrhea. The patient started to have symptoms after treatment of amoeba infection for nearly 40 days, and there was no electrolyte imbalance or hypoglycemia. Thus, amoebic infection and metabolic disturbance were not considered as secondary factors. Considering the metronidazole history, we assumed that MIE was the cause of RESLES, though the etiology of EBV infection could not be ruled out. After cessation of metronidazole, the lesion disappeared on follow-up MRI. Therefore, MIE was diagnosed eventually.

MIE is a rare adverse effect of metronidazole. Metronidazole, a nitroimidazole, is widely used against microaerophiles including *Helicobacter pylori*, and protozoa including *Entamoeba histolytica* (amoebic dysentery) and *Giardia lamblia* (giardiasis). Metronidazole also remains a good choice for colitis associated with *Clostridium difficile*, and for infections of the oral cavity, such as periodontitis.^[1]^ Metronidazole is generally well tolerated, but in rare cases, MIE has been reported. Liver disease is the most common pre-existing condition. There are varying reports of the effects of cumulative dose and duration of therapy on MIE.^[7]^ The manifestations of MIE mainly include dysarthria, gait instability, limb dyscoordination, altered mental status, and seizures.^[4,7]^ Some studies have proposed the triad of MIE, involving altered mental status, convulsive seizures, and cerebellar dysfunction.^[7]^ The most common lesion sites are the bilateral dentate nucleus of the cerebellum (up to 90%),^[8]^ midbrain, corpus callosum, pons, medulla, and subcortical white matter.^[4,9]^ Typical lesions in MRI show symmetrical T2-weighted or fluid attenuation inversion recovery hyperintensity, with minimal hypointensity on T1-weighted images. High signals on DWI are always found, which are most frequently located in the corpus callosum, while apparent diffusion coefficient values are variable.^[2]^

The majority of patients can partially or completely recover after stopping metronidazole. However, in rare cases, metronidazole can cause serious sequelae, and even death.^[10]^ Lesions of the cerebral white matter may be associated with a worse prognosis. However, there is no definitive relationship between the cumulative dose of metronidazole and patient outcome.^[4,7]^ The key point is early diagnosis and early cessation of metronidazole. In the present case, the patient developed amoebic dysentery on the basis of ulcerative colitis after eating undercooked food, while Epstein–Barr virus infection cannot be ruled out. He presented with speech difficulties, and MRI showed isolated involvement of the splenium of the corpus callosum. After cessation of metronidazole for 1.5 months, the lesion totally disappeared.

Wernicke's encephalopathy needs to be differentiated in the present case. In addition to central nervous system dysfunction, Wernicke's encephalopathy can cause PN, although is mainly associated with thiamine deficiency because of alcohol abuse and malnutrition. The Wernicke triad consists of an acute confusion, ataxia, and ophthalmoplegia, but has a low sensitivity. Characteristic features of MRI are bilateral and symmetrical T2 hyperintensities in typical (thalami, mammillary bodies, tectal plate, and periaqueductal area) and atypical (cerebellum, cranial nerve nuclei, and cerebral cortex) areas.^[11]^ However, our patient did not have a history of alcohol abuse, and no typical manifestations or typical lesion areas. Therefore, Wernicke's encephalopathy was ruled out. Further, although the patient took other drugs such as etimicin, meropenem, and mesalazine, the use of these drugs and his symptoms were not closely linked chronologically.

Based on his symptoms and results of electrophysiological examination, bilaterally symmetric sensory PN was diagnosed, involving both small-fiber and large-fiber peripheral nerves. These findings are consistent with features of toxic PN. Considering the metronidazole history and concomitant MIE, metronidazole-associated PN was diagnosed. Compared with MIE, metronidazole-associated PN is more common, though the overall incidence remains unclear. A review reported that most cases of metronidazole associated PN are seen in patients with >42 g total dose or >4 weeks of metronidazole treatment. The majority of patients can recover after stopping metronidazole, although the time to symptom improvement varies widely. Moreover, for unknown reasons a minority of patients do not recover.^[3]^

The mechanism of metronidazole-associated PN remains unknown. In experimental studies, Bradley et al. found that metronidazole or its metabolic products may bind to RNA, inhibit neuronal protein synthesis, and cause peripheral axonal degeneration.^[12]^ Alston et al. also reported that the adverse effects of metronidazole were related to, at least in part, its conversion to a thiamine analog and consequent vitamin B1 antagonism,^[13]^ resulting in dystrophic PN. In the present study, the patient developed neurotoxicity with a cumulative metronidazole dose >42 g, consistent with previous reports. Electrophysiological examination also showed characteristics of axonal degeneration, signifying a poor prognosis, which may explain his inability to live normally at approximately 6 months after cessation of metronidazole.

Our case was characterized by both central neurotoxicity and PN caused by metronidazole. In a recent review of the literature, almost one-third of MIE patients were found to present with concomitant metronidazole-induced polyneuropathy,^[7]^ while an isolated lesion of the corpus callosum in MIE is quite rare. We have summarized all previously reported cases^[2,14,15]^ in Table 1. Interestingly, all patients developed subjective symptoms or objective evidence of PN. Thus, we speculate that there is a relationship between RESLES caused by MIE and metronidazole-associated PN, which warrants further validation. In contrast to our case, all other reported cases had a good prognosis for PN (apart from 1 case in which prognosis was not described). Further, with respect to MIE, only 1 case exhibited continuous existence of the lesion, although the patient improved symptomatically.

**Table 1:**
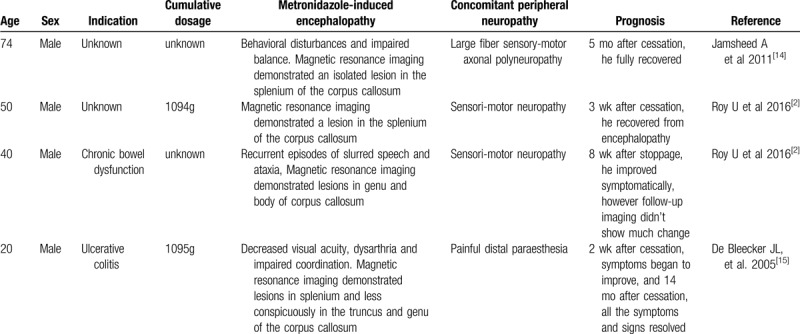
Cases of isolated involvement of the corpus callosum in metronidazole-induced encephalopathy.

The pathogenic mechanism of neurotoxicity caused by metronidazole remains unknown, although a number of mechanisms have been proposed. Mason reported that metronidazole can be reduced by catecholamine neurotransmitters, and that free radicals are formed in the process, which can cause neurotoxicity.^[16]^ Evans et al. also reported that dogs with metronidazole toxicosis recovered faster when treated with diazepam, and suggested that this may relate to modulation of gamma-aminobutyric acid receptors within the cerebellar and vestibular systems.^[17]^ In multiple reported MIE cases, there is evidence of a high signal on DWI and multiple signals on apparent diffusion coefficient, indicating both cytotoxic and vasogenic edema, which may result from the toxic effects of metronidazole.^[4]^ Future experimental and clinical studies are required to clarify the mechanisms of metronidazole-induced neurotoxicity.

In summary, we present a patient with MIE with isolated corpus callosum involvement and metronidazole associated PN, which fully recovered after cessation of metronidazole. Thus, in patients with RESLES, MIE needs to be considered if the patient has a history of metronidazole medication, especially when presenting with concomitant PN.

## Acknowledgments

The authors thank the patient for his contributions to this work, and Liwen Bianji, Edanz Editing China, for editing the English text of a draft of this manuscript.

## Author contributions

**Conceptualization:** Qing Peng.

**Data curation:** Qian You, Jing Zhang, Shui Liu.

**Investigation:** Qing Peng, Qian You, Shui Liu.

**Methodology:** Qian You, Jing Zhang, Shui Liu.

**Resources:** Qing Peng.

**Supervision:** Qing Peng.

**Writing – original draft:** Qian You, Jing Zhang.

**Writing – review and editing:** Qing Peng.

## References

[1] Dingsdag SA Hunter N Metronidazole: an update on metabolism, structure-cytotoxicity and resistance mechanisms. J Antimicrob Chemother 2018;73:265–79. 29077920 10.1093/jac/dkx351

[2] Roy U Panwar A Pandit A Clinical and neuroradiological spectrum of metronidazole induced encephalopathy: our experience and the review of literature. J Clin Diagn Res 2016;10:OE01–9. 10.7860/JCDR/2016/19032.8054 PMC4963700 27504340

[3] Goolsby TA Jakeman B Gaynes RP Clinical relevance of metronidazole and peripheral neuropathy: a systematic review of the literature. Int J Antimicrob Agents 2018;51:319–25. 28887203 10.1016/j.ijantimicag.2017.08.033

[4] Sorensen CG Karlsson WK Amin FM Metronidazole-induced encephalopathy: a systematic review. J Neurol 2018. 10.1007/s00415-018-9147-6 30536109

[5] Feraco P Porretti G Marchio G Mild encephalitis/encephalopathy with reversible splenial lesion (MERS) due to cytomegalovirus: case report and review of the literature. Neuropediatrics 2018;49:68–71. 29179234 10.1055/s-0037-1608779

[6] Yuan J Yang S Wang S Mild encephalitis/encephalopathy with reversible splenial lesion (MERS) in adults-a case report and literature review. BMC Neurol 2017;17:103. 28545419 10.1186/s12883-017-0875-5 PMC5445341

[7] Kuriyama A Jackson JL Doi A Metronidazole-induced central nervous system toxicity: a systematic review. Clin Neuropharmacol 2011;34:241–7. 21996645 10.1097/WNF.0b013e3182334b35

[8] Yoon Andrew Cho-Park FDLP Tracey A Milligan Cho Radiographic evolution of a rapidly reversible leukoencephalopathy due to metronidazole. Neurology: Clinical Practice 2013;272–4. 29473640 10.1212/CPJ.0b013e318296f098 PMC5798518

[9] Seong Soo Lee S-HC Seung Young Lee Chang June Song Reversible inferior colliculus lesion in metronidazole-induced encephalopathy: magnetic resonance findings on diffusion-weighted and fluid attenuated inversion recovery imaging. J Comput Assist Tomogr 2009;33:305–8. 19346865 10.1097/RCT.0b013e31817e6f58

[10] Onuma Y Oki M Komatsu M Irreversible metronidazole encephalopathy in an elderly woman with primary biliary cholangitis. J Gen Fam Med 2017;18:436–8. 29264081 10.1002/jgf2.91 PMC5729387

[11] Manzo G De Gennaro A Cozzolino A MR imaging findings in alcoholic and nonalcoholic acute Wernicke's encephalopathy: a review. Biomed Res Int 2014;2014:503596. 25050351 10.1155/2014/503596 PMC4094710

[12] Bradley WG Rassol KI CG Metronidazole neuropathy. Br Med J 1977;2:610–1. 198056 10.1136/bmj.2.6087.610 PMC1631560

[13] Abeles TAARH Enzymatic conversion of the antibiotic metronidazole to an analog of thiamine. Arch Biochem Biophys 1987;257:357–62. 2821910 10.1016/0003-9861(87)90577-7

[14] Jamsheed A Desai JD Michel M Metronidazole-induced encephalopathy: case report and review of MRI findings. Can J Neurol Sci 2011;00:512–3. 10.1017/s0317167100011951 21515514

[15] De Bleecker JL Leroy BP Meire VI Reversible visual deficit and Corpus callosum lesions due to metronidazole toxicity. Eur Neurol 2005;53:93–5. 15855780 10.1159/000085506

[16] Mason DNRRRP Generation of nitro radical anions of some 5-nitrofurans, 2- and 5-nitroimidazoles by norepinephrine. J Biol Chem 1987;262:11731–6. 2887562

[17] Jason Evans DL Kim K Randy L Diazepam as a treatment for metronidazole toxicosis in dogs: a retrospective study of 21 cases. J Vet Intern Med 2003;17:304–10. 12774970 10.1111/j.1939-1676.2003.tb02452.x

